# Supplementary material for: Targeting HR Repair as a Synthetic Lethal Approach to Increase DNA Damage Sensitivity by a RAD52 Inhibitor in BRCA2-Deficient Cancer Cells
Source: Int J Mol Sci. 2021 Apr 23;22(9):4422. doi: 10.3390/ijms22094422 (PMC8122931; doi:10.3390/ijms22094422)
Supplement: Supplementary file 1 [file ijms-22-04422-s001.zip › ijms-1188323-supplementary.pdf]

Supplementary Materials

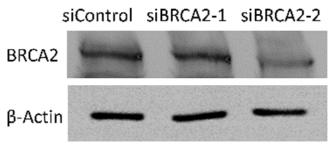

Figure S1. The inhibition of BRCA2 expression by different siRNAs in MCF7 cells

MCF7 cells were transfected with siControl, siBRCA2-1 and siBRCA2-2 for 48 hours. The different siRNA clones were harvested and subjected to western blotting against BRCA2.

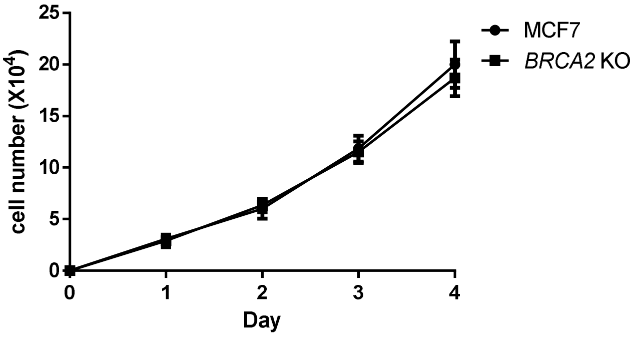

Figure S2. The proliferation of MCF7 and BRCA2-knockout MCF7 cells

MCF7 or BRCA2-knockout MCF7 cells were seeded at $2X10^4$ cells in 48-well plates, and every 24 hours, the cells were resuspended, and the cell number was counted. After counting each group, the cells were placed back to the 48-well plate.

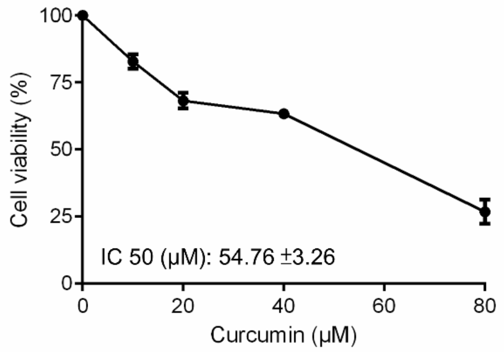

Figure S3. Effects of curcumin on the viability of MCF7 cells. Cells were treated with various concentrations of curcumin for 24 h and the viability of treated cells was evaluated by MTT assay. Data are expressed as mean ± SD of three independent experiments.

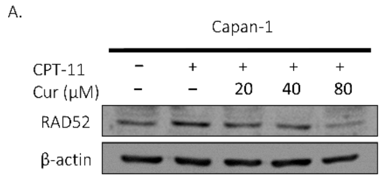

Figure S4. Curcumin inhibits the protein expression of RAD52 recombinase following CPT-11 treatment in Capan1 cells. Capan1 cells were treated with 5 μM CPT-11 combined with the indicated curcumin for 8 hours. Cell samples were taken and processed for immunoblot analysis using an anti-RAD52 antibody.
